# PIN1 and PIN4 inhibition *via* parvulin impeders Juglone, PiB, ATRA, 6,7,4′-THIF, KPT6566, and EGCG thwarted hepatitis B virus replication

**DOI:** 10.3389/fmicb.2023.921653

**Published:** 2023-01-25

**Authors:** Umar Saeed, Zahra Zahid Piracha

**Affiliations:** Department of Medical Research and Development, International Center of Medical Sciences Research (ICMSR), Islamabad, Pakistan

**Keywords:** PIN1 and PIN4, HBV, Juglone, PiB, ATRA, KPT6566, EGCG, THIF

## Abstract

**Introduction:**

Human parvulin peptidyl prolyl cis/trans isomerases PIN1 and PIN4 play important roles in cell cycle progression, DNA binding, protein folding and chromatin remodeling, ribosome biogenesis, and tubulin polymerization. In this article, we found that endogenous PIN1 and PIN4 were upregulated in selected hepatocellular carcinoma (HCC) cell lines.

**Methods:**

In this study, we inhibited PIN1 and PIN4 *via* parvulin inhibitors (Juglone, PiB, ATRA, 6,7,4′-THIF, KPT6566, and EGCG). The native agarose gel electrophoresis (NAGE) immunoblotting analysis revealed that upon PIN1 and/ or PIN4 inhibition, the HBc protein expression and core particle or capsid synthesis reduced remarkably. The effects of PIN4 inhibition on hepatitis B virus (HBV) replication were more pronounced as compared to that of PIN1. The Northern and Southern blotting revealed reduced HBV RNA and DNA levels.

**Results:**

During the HBV course of infection, Juglone, PiB, ATRA, 6,7,4′-THIF, KPT6566, and EGCG-mediated inhibition of PIN1 and PIN4 significantly lowered HBV transcriptional activities without affecting total levels of covalently closed circular DNA (cccDNA). Similar to the inhibitory effects of PIN1 and PIN4 on HBV replication, the knockdown of PIN1 and PIN4 in HBV infection cells revealed significantly reduced amounts of intracellular HBc, HBs, HBV pgRNA, SmRNAs, core particles, and HBV DNA synthesis. Similarly, PIN1 and PIN4 KD abrogated extracellular virion release, naked capsid levels, and HBV DNA levels. In comparison with PIN1 KD, the PIN4 KD showed reduced HBc and/or core particle stabilities, indicating that PIN4 is more critically involved in HBV replication. Chromatin immunoprecipitation (ChIP) assays revealed that in contrast to DNA binding PIN4 proteins, the PIN1 did not show binding to cccDNA. Similarly, upon PIN1 KD, the HBc recruitment to cccDNA remained unaffected. However, PIN4 KD significantly abrogated PIN4 binding to cccDNA, followed by HBc recruitment to cccDNA and restricted HBV transcriptional activities. These effects were more pronounced in PIN4 KD cells upon drug treatment in HBV-infected cells.

**Conclusion:**

The comparative analysis revealed that in contrast to PIN1, PIN4 is more critically involved in enhancing HBV replication. Thus, PIN1 and PIN4 inhibition or knockdown might be novel therapeutic targets to suppress HBV infection. targets to suppress HBV infection.

## Introduction

Human hepatitis B virus (HBV) possesses exclusive tropism for hepatocytes (Seeger and Mason, 2015). Despite the availability of HBV vaccines, HBV has remained a huge burden on global public health, causing acute hepatitis, which may further develop into chronic HBV or may progress toward hepatocellular carcinoma (HCC; Seeger and Mason, 2015). The chronic hepatitis B (CHB) infection affected the lives of 257 million people worldwide. On average, CHB infection causes approximately 0.88 million deaths per year (Revill et al., 2020).

Hepatitis B virus replication mechanisms are well known; however, the effects of several host cellular proteins that support HBV replication are not fully understood. Contemplating HBV biology at a cellular or molecular level is important for designing novel therapeutic agents against viral infection. Sodium/bile acid cotransporter or Na^+^ − taurocholate cotransporting polypeptide (NTCP) or liver bile acid transporter (LBAT) is a protein surface receptor that is essential for HBV entry to hepatocytes. The NTCP/LBAT is encoded by the solute carrier family 10 member 1 gene (*SLC10A1*; Yan et al., 2012). After entering the cell, the HBV capsids or core particles containing partially double-stranded, relaxed circular (RC) DNA are delivered into the nucleus and release RC DNA. In the nucleus, the polymerase-bound RC DNA alters into chromatin-like conformation called covalently closed circular DNA (cccDNA), which generate HBV transcripts. The cccDNA transcribes into pre-genomic RNA (3.5 kb) pgRNA, 0.7 kb × mRNAs, and sub-genomic (2.4 and 2.1 kb) S mRNAs (Nassal, 2015; Seeger and Mason, 2015; Lucifora and Protzer, 2016). A single copy of cccDNA in the liver is capable of re-initiating full boost HBV infection (Nassal, 2015; Lucifora and Protzer, 2016).

Peptidyl-prolyl-cis/trans isomerases (PPIases) are a vast array of enzymes capable of regulating cis/trans isomerization, therefore assisting in the folding and functions of target proteins (Gothel and Marahiel, 1999; Lu et al., 2007). The PPIase proteins are grouped into four families, including FK506-binding proteins, cyclophilins, protein Ser/Thr phosphatase-2A (PP2A)-activator (PTPA), and parvulins (Lu et al., 2007). Two parvulin genes are identified in the human genome including PIN1 and PIN4 (Lu et al., 1996; Rulten et al., 1999; Mueller et al., 2006). The PIN1 gene generates the PIN1 protein, while the PIN4 gene generates parvulin 14 (Par14) and/or 17 (Par17) proteins (Mueller et al., 2006). The PIN1 possesses 163 amino acids constituting two important domains, namely, WW and PPIase domains. Par14 consists of 131 amino acids, while its other isoform, produced by alternative transcription initiation, i.e., Par17, is composed of 156 amino acids. The N-terminal of Par17 contains additional 25 amino acids that constitute amphipathic α-helix (Uchida et al., 2003; Mueller et al., 2006). The PIN1 and PIN4 have been involved in interacting with HBx and HBV core particles. The parvulin proteins regulate ribosomal RNA processing, DNA binding, chromatin remodeling, tubulin polymerization, and cell cycle progression (Thiele et al., 2011; Saningong and Bayer, 2015; Matena et al., 2018).

**Figure 1:**
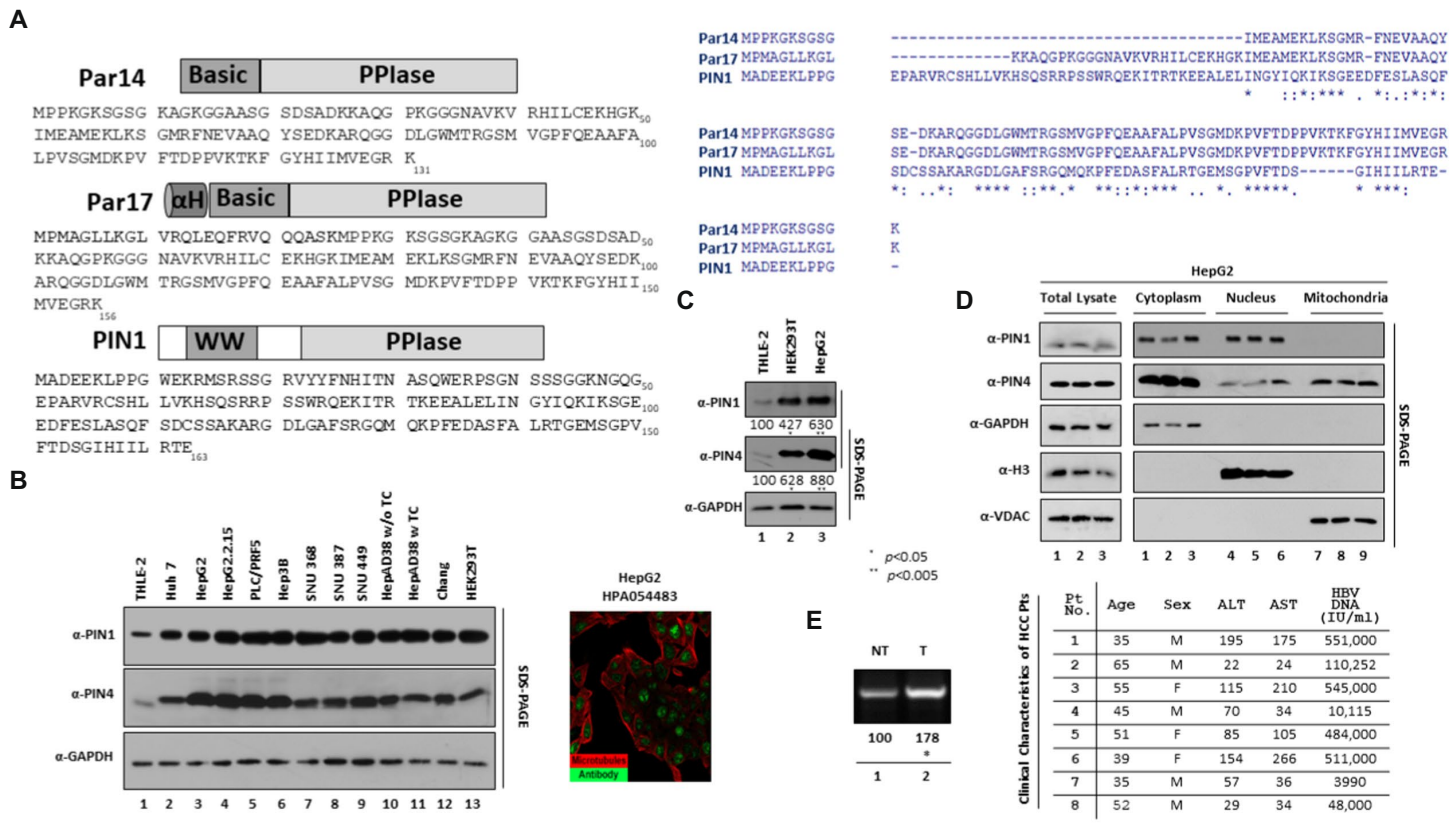
Endogenous expression of PIN1 and PIN4 (Par14/Par17) in selected HCC cells. **(A)** Sequence of PIN1 and PIN4 (Par14 and Par17) proteins. The PIN1 protein is comprised of 163 amino acids constituting two important domains, WW and PPIase domains. The PIN4-encoded Par14 is comprised of 131 amino acids, which form the N-terminal DNA binding Basic domain and PPIase domain. Similar to Par14, the PIN4-encoded Par17 has additional 25 amino acids alpha-helical domain prior to Basic and PPIase domains. Amino acid sequence analysis of PIN1, Par14, and Par17 was made by ClustalW2. **(B)** Selected HCC cell lines express higher levels of endogenous PIN1, and PIN4 proteins than THLE-2 immortalized human liver epithelial cells. THLE-2 (lane 1), Huh7 (lane 2), HepG2 (lane 3), HepG2.2.15 (lane 4), PLC/PRF5 (lane 5), Hep3B (lane 6), SNU368 (lane 7), SNU387 (lane 8), SNU449 (lane 9), HepAD38 in TC-containing (lane 11) or TC-depleted media (lane 10), Chang (lane 11), and HEK293T (lane 12) cells were plated at equal densities (2 × 10^6^ cells per 6 cm plate). Human Protein Atlas PIN4 expression in HepG2 is shown with reference-ID HPA054483. **(C)** Relative PIN4 gene product levels in THLE-2, HEK293T, and HepG2 cells. THLE-2 (lane 1), HEK293T (lane 2), and HepG2 cells (lane 3) were seeded as described above. At 72 h post-seeding, equal quantities of protein (as determined by Bradford Assay) from cell lysates, as described previously (Piracha et al., 2018). **(D)** PIN1 is localized to cytoplasm and nucleus, while PIN4 protein was localized in nuclear, cytoplasmic, and mitochondrial regions. HepG2 cells were seeded in 6 cm plates in triplicate (lanes 1–3). At 72 h post-transfection, the cellular fractions were prepared as described in the “Material and Methods” section. SDS-PAGE and immunoblotting (with mouse monoclonal anti-PIN1, rabbit monoclonal anti-PIN4, rabbit polyclonal anti-Myc, mouse monoclonal anti-GAPDH, rabbit polyclonal anti-H3, and mouse monoclonal anti-VDAC antibodies) were performed. **(E)** cccDNA levels in paired tumor and adjacent nontumor liver biopsy samples from patients with HBV-HCC. Cellular lysates from biopsy samples were prepared in the weight per volume w/v ratio in the mPER buffer, as described previously (Piracha et al., 2020). cccDNA levels were determined from clinical samples using cccDNA-specific primers as described in the “Material and Methods” section. The clinical parameters of the patients including Age, Sex, ALT, AST, and HBV DNA (IU/ml) are shown. Data are shown as representations from independent three experiments. The statistical significance values were determined using Student’s *t*-test. *p < 0.05, **p < 0.005 relative to control.

Several host PPIases support viral protein modifications and replications. Feline coronavirus replication has been reported to be affected by both cyclophilin A and cyclophilin B (Tanaka et al., 2006). FKBP8 interacts with the NS5A protein of the swine fever virus to promote viral replication (Li et al., 2016). The significance of FKBP6 toward the replication of the hepatitis C virus (HCV) has also been identified (Kasai et al., 2015). HCV NS5A has been reported as a substrate for the PPIase activity of cyclophilins A and B, while cyclophilin B had already been reported to be critical for HCV replication (Watashi et al., 2005; Hanoulle et al., 2009). PIN1 plays important roles in the replication of various viruses such as HBV, herpesvirus, Epstein–Barr virus (EBV), feline coronavirus, HIV, and HCV (Milbradt et al., 2016; Li et al., 2021). Mutational studies illustrated that Par14 phosphorylation on Ser19 regulates the ability to bind DNA and leads to sub-cellular localization to the nucleus (Reimer et al., 2003). Human Par14 was reported in the exosomes originating from K-RAS collateral tumor cells (Demory Beckler et al., 2013). It has been reported that Par14 correlates with the occurrence of autoimmune chronic cholestatic liver disease and primary biliary cirrhosis (PBC), which may potentially cause cholestatic fibrosis and/or cirrhosis (Mitchell et al., 2011). It has been reported that PIN4 may contribute to higher invasiveness of recipient cancer cells (Higginbotham et al., 2011). Human Protein Atlas showed elevated expression of parvulins in HCC HepG2 cell lines (Figure 1).

Juglone (also known as 5-hydroxyl-1,4-naphthoquinone) inactivates or inhibits PIN1 and PIN4 as a competitive irreversible inhibitor (Hennig et al., 1998; Chao et al., 2001). PiB (or 1,3,6,8-tetrahydro-1,3,6,8-tetraoxo-benzo [lmn][3,8] phenanthroline-2,7-diacetic acid, 2,7-diethyl ester) is a competitive reversible inhibitor of the PIN1 and PIN4 (Uchida et al., 2003). All-trans-retinoic acid (ATRA) also known as tretinoin or vitamin A acid is a potent parvulin PIN1 inhibitor. ATRA inhibits PIN1 activity by directly binding to the catalytic PPIase domain (Hennig et al., 1998). 6,7,4′-Trihydroxyisoflavone (6,7,4’-THIF), 6,7-dihydroxy-3-(4-hydroxyphenyl)-4H-chromen-4-one, or demethyltexasin effectively suppresses the isomerase activity of PIN1, thus acting as a potential parvulin PIN1 inhibitor (Lim et al., 2017; Chuang et al., 2021). The KPT6566 or 2-[[4-[[[4-(1,1-dimethylethyl)phenyl]sulfonyl]imino]-1,4-dihydro-1-oxo-2-naphthalenyl]thio] acetic acid blocks its PPIase activity and causes PIN1 degeneration (Campaner et al., 2017; Yu et al., 2020). Epigallocatechin gallate (EGCG), also known as-cis-2-(3,4,5-trihydroxyphenyl)-3,4-dihydro-1(2H)-benzopyran-3,5,7-triol 3-gallate or-cis-3,3′,4′,5,5′,7hexahydroxy-flavane-3-gallate, directly binds with PIN1 and consequently inhibits its PPlase activity, thus acting as a potent PIN1 inhibitor (Della Via et al., 2021).

Herein, we aimed to determine the inhibitory roles of PIN1 and PIN4 through parvulin inhibitors, namely, Juglone, PiB, ATRA, 6,7,4′-THIF, KPT6566, and EGCG. Interestingly, upon the inhibition of parvulins, the expression of HBc, HBc recruitment to HBV cccDNA, HBV transcriptional activities, core particle synthesis, HBV DNA synthesis, and HBV virion secretion was significantly abrogated. In comparison with PIN1, upon PIN4 KD followed by Juglone, PiB, ATRA, 6,7,4′-THIF, KPT6566, and EGCG treatment, the inhibitory effects on HBV replication were even more pronounced.

## Results

### Human parvulins are highly expressed in HBV replicating and non-replicating hepatocellular carcinoma cells

The human genome comprises two parvulin genes, namely, peptidyl-prolyl cis-trans isomerase NIMA-interacting 1 (*PIN1*) that encodes Pin1 protein, and peptidyl-prolyl cis-trans isomerase NIMA-interacting 4 (*PIN4*) that encodes Par14 and Par17 proteins (Kessler et al., 2007). The anti-PIN4 antibody can detect both protein isoforms Par14 and Par17 (Figure 1A). Human Protein Atlas revealed paramount expression of parvulin mRNAs in the liver. We examined the endogenous expression of selected parvulin proteins in human HBV-replicating and non-replicating HCC cell lines by comparing with immortalized liver transformed human liver epithelial-2 (THLE-2) cells (simian virus 40 large tumor antigen-immortalized normal human liver epithelial cells that express hepatocyte characteristics). The Huh7, HepG2, PLC/PRF5, Hep3B, SNU 368, SNU 387, and SNU 449 HCC cell lines and HBV replicating HepG2.2.15 and HepAD38 cell lines revealed a higher expression of PIN1 and PIN4 proteins (Figure 1B). Since HEK 293 T cells are widely applied for the propagation of adenovirus vectors, we also included HEK 293 T to determine indigenous protein levels of parvulin proteins in comparison with normal THLE-2 cells. Of note, the expression levels of PIN1 and PIN4 were significantly higher in HepG2 and HEK 293 T than in THLE-2 cells (Figure 1C, top panel, lane 1 vs. 2–13 and second panel, lane 1 vs. 2–13).

Furthermore, we determined the physical localization of endogenous PIN1 and PIN4 proteins in the cell. Through differential centrifugation technique, cellular fractions (cytoplasm, nucleus, and mitochondria) were prepared from HepG2 cells. The PIN1 localized in cytoplasmic and nuclear fractions (Figure 1D top panel, lanes 1–3 and lanes 4–6), while PIN4 showed localization in the cytoplasm, nuclei, and mitochondrial regions (Figure 1D second panel, lanes 1–3, lanes 4–6, and lanes 7–9).

Since the endogenous PIN1 and PIN4 levels remained higher in HBV replicating and non-replicating HCC-derived cell lines, we examined PIN1 and PIN4 levels in the biopsied tumor and non-tumor adjacent liver tissues obtained from eight patients of HBV-associated HCC. However, PIN1 and PIN4 expression levels remained unchanged. The HBV DNAs from patients 1, 2, 3, and 4 with HCC were identified as 551,000, 110,252, 545,000, and 10,115 IU/ml, respectively. In contrast, for HBV-HCC patients 5, 6, 7, and 8 were 484,000, 511,000, 3,990, and 48,000 IU/ml, respectively (Figure 1E). From the clinical specimens, HBV cccDNA levels were examined, as described previously (Zhang et al., 2017). Of note, the cccDNA levels were higher in HBV-associated biopsied tumors than in the nontumor liver tissues (Figure 1E, lane 1 vs. 2).

### The optimum dosage of parvulin inhibitors in HCC cell lines

**Figure 2:**
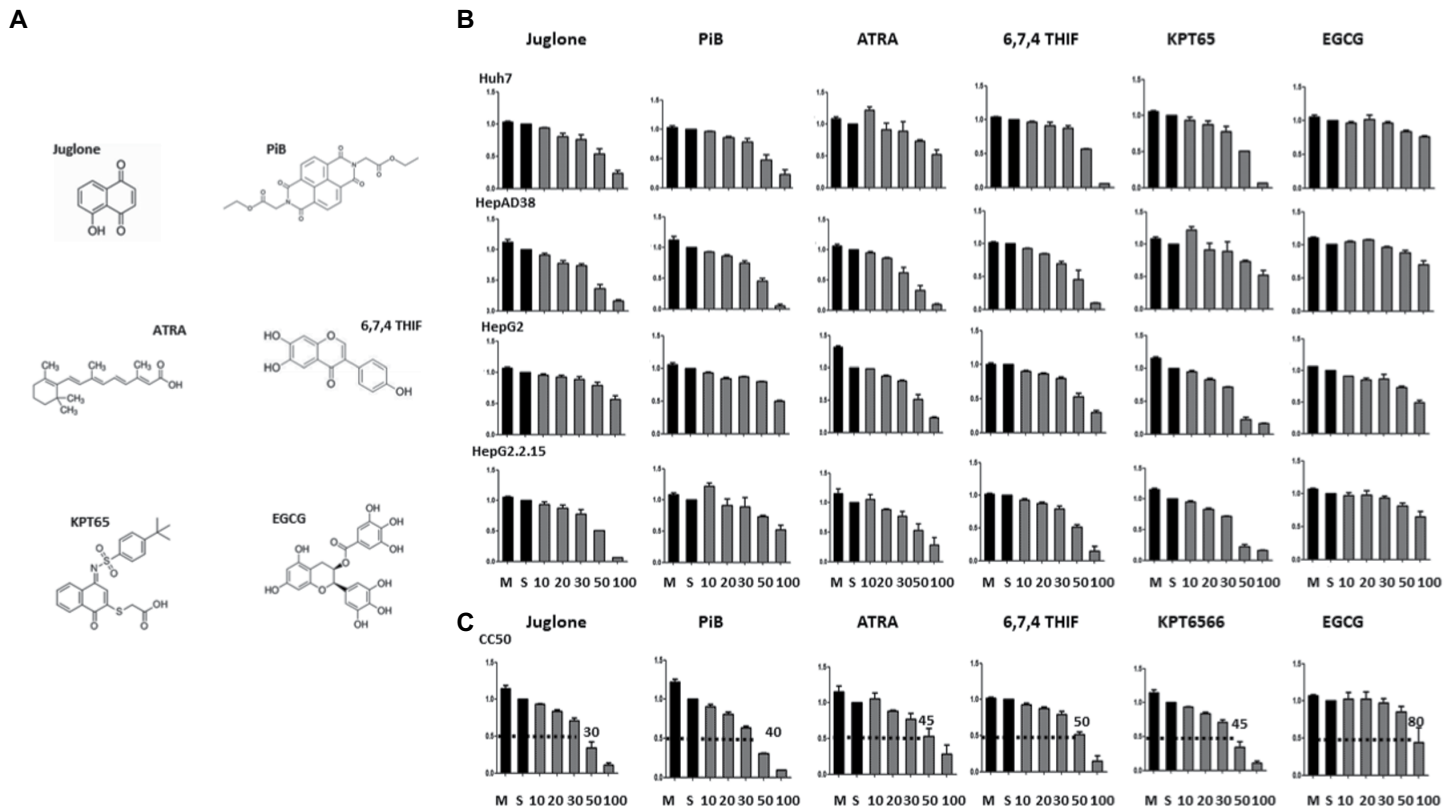
MTT assay to measure the cytotoxicity of parvulin inhibitors (Juglone, PiB, ATRA, 6,7,4′-THIF, KPT6566, and EGCG). **(A)** The chemical structures of parvulin inhibitors including Juglone, PiB, ATRA, 6,7,4′-THIF, KPT6566, and EGCG are shown. **(B)** Huh7, HepAD38, HepG2, and HepG2.2.15 cells were exposed to varying concentrations (10 μM, 20 μM, 30 μM, 50 μM, and 100 μM) of inhibitors Juglone, PiB, ATRA, 6,7,4′-THIF, KPT6566, and EGCG for 48 h and values were determined *via* MTT assay. **(C)** The concentrations of Juglone, PiB, ATRA, 6,7,4′-THIF, KPT6566, and EGCG at which cell viability was reduced to 50% (CC50) to that of the control (cells treated by the solvent) were determined.

Since PIN1 and PIN4 were highly expressed in HCC cells, we speculated that the inhibition of parvulins *via* Juglone, PiB, ATRA, 6,7,4′-THIF, KPT6566, and EGCG might affect HBV replication (Figure 2A). The optimum dosage was determined in several HCC cell lines. Compared to mock or solvent-treated 10, 20, 30, 50, or 100 μM, drug-treated cell viabilities were determined by the MTT assay in Huh7, HepAD38, HepG2, and HepG2.2.15 cells (Figure 2B). The data from triplicate experiments were used to examine 50% cytotoxic concentration (CC50) of Juglone, PiB, ATRA, 6,7,4′-THIF, KPT6566, and EGCG *via* the GraphPad Prism software (Figure 2C). The CC50 values for Juglone and PiB were 30 and 40 μM, respectively. In contrast, 45 and 50 μM of ATRA and 6,7,4′-THIF showed 50% cytotoxic effects. However, KPT6566 and EGCG depicted 45 and 80 μM as CC50 values, respectively. We optimized the effects of Juglone and PiB parvulin inhibitors in diverse HBV replicating HCC cell lines at 20 μM concentration. In addition, 30 μM concentrations were used to determine the effects of ATRA, 6,7,4′-THIF, and KPT6566 on HBV replication. While a 40 μM concentration of EGCG was optimized for determining the effects on HBV replication in multiple HCC cell lines.

### The Juglone, a competitive irreversible inhibitor of PIN1 and PIN4, abrogated expression of HBc, HBV transcription, core particle levels, and consequently HBV replication

**Figure 3:**
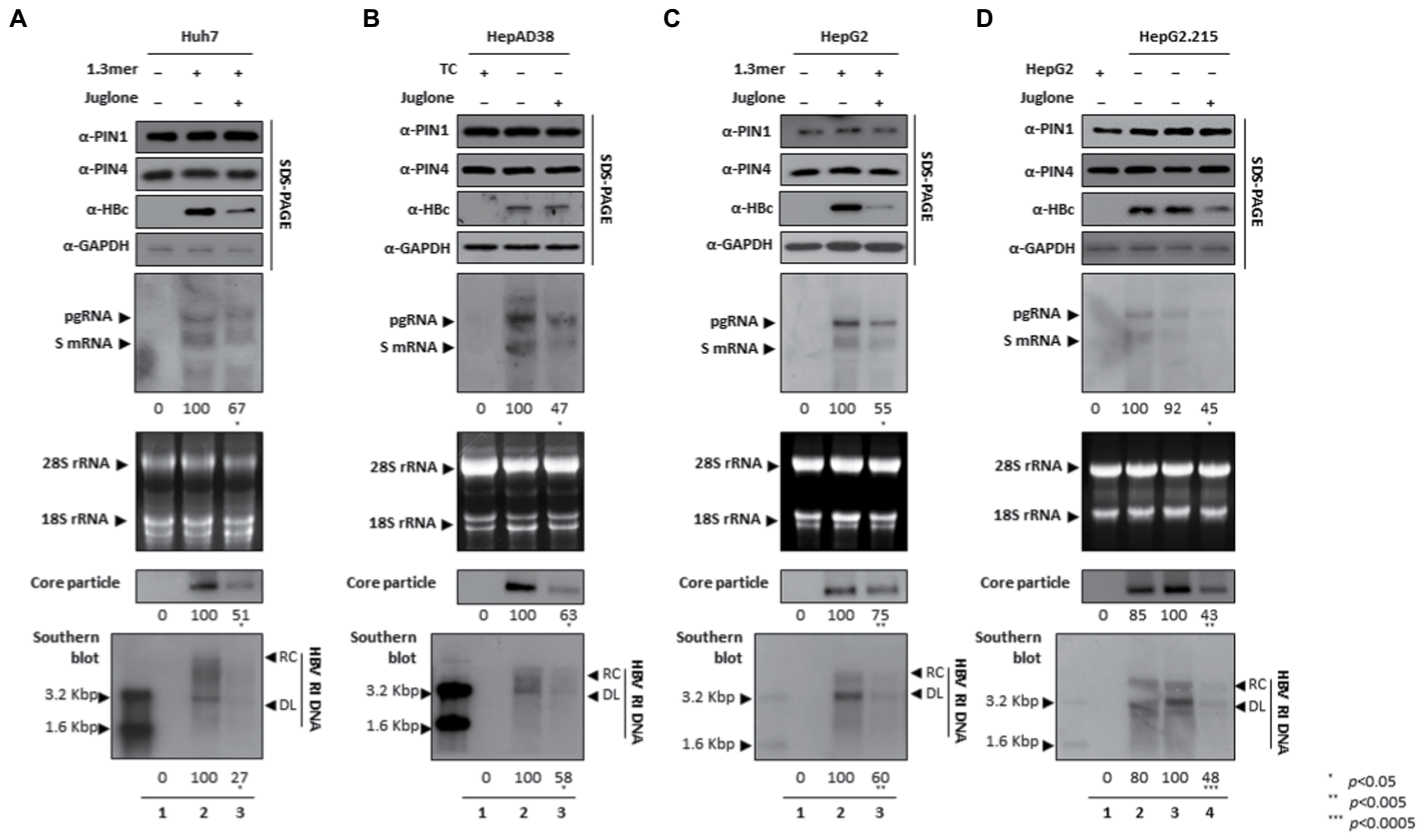
Inhibition of PIN1 and PIN4 by Juglone reduces replication of HBV in Huh7, HepAD38, HepG2, and HepG2.2.15. **(A–D)** Juglone reduces HBV replication. **(A)** The Huh7 cells were mock-transfected (lane 1), transfected with 4 μg of 1.3 mer HBV WT and treated with ethanol (lane 2), or transfected by 1.3 mer HBV WT (4 μg) and treated with 20 μM of Juglone for 72 h (lane 3). **(B)** HepAD38 was treated with mock (none, lane 1) or ethanol (lane 2) or treated with 20 μM Juglone (lane 3) for 72 h. **(C)** HepG2 cells were transfected with mock (lane 1), 1.3 mer HBV WT (4 μg) transfected and treated with ethanol (lane 2), or 4 μg 1.3 mer HBV WT transfected and treated with Juglone (20 μM) for 72 h (lane 3). **(D)** HepG2.2.15 cells were treated with mock (none, lane 2) or treated ethanol (lane 3) or Juglone (20 μM) treated for 72 h (lane 4). Lane 1 indicates mock-transfected HepG2 cells (negative control). The lysates were subjected to SDS-PAGE and immunoblotting, NAGE, Northern, and Southern blotting, as described previously (Piracha et al., 2018). The presented data are representative of three independent experiments. The statistical analysis was determined using Student’s *t*-test. *p < 0.05, **p < 0.005, ***p < 0.0005 relative to the control.

Since parvulins were highly expressed in HCC cells, we speculated that parvulins gene and/or gene products might affect HBV replication. Juglone-mediated inhibition of PIN1 and PIN4 effects on HBV replication was determined in Huh7, HepAD38, HepG2, and HepG2.2.15 HCC cell lines. The expressions of HBc, core particle synthesis, and HBV DNA levels were dramatically reduced. From the downregulated HBc expression by PIN1 and PIN4 inhibition *via* Juglone, we speculated that Juglone-mediated inhibition might affect the transcriptional activities of HBV. To examine the HBV transcription, Northern blotting was performed. The levels of HBV RNAs were significantly decreased in Juglone-treated 1.3 mer HBV-WT transfected Huh7 (Figure 3A), HepAD38 (Figure 3B), 1.3 mer HBV-WT transfected HepG2 (Figure 3C), and HepG2.2.15 (Figure 3D) cells (Figures 3A–C lanes 2 and 3, Figure 3D lanes 3 and 4).

### The PiB, a competitive reversible inhibitor of PIN1 and PIN4, and restricted HBV replication

**Figure 4:**
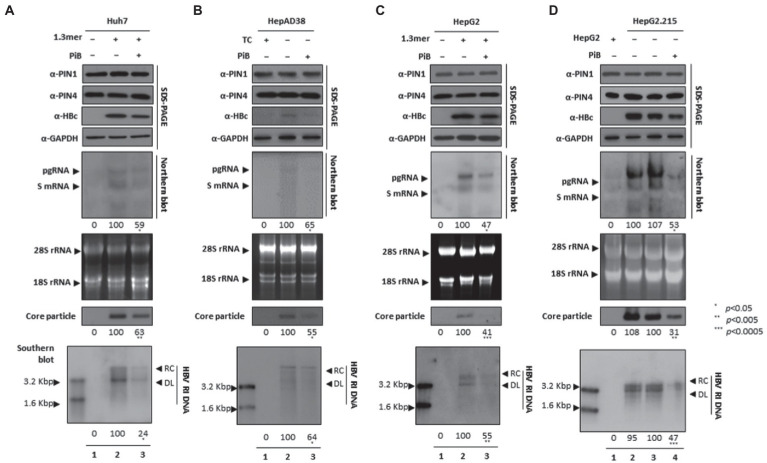
PiB-mediated inhibition of PIN1 and PIN4 reduces HBV replication in Huh7, HepAD38, HepG2, and HepG2.2.15. **(A–D)** PiB reduces HBV replication. **(A)** The Huh7 cells were mock-transfected (lane 1), transfected with 4 μg of 1.3 mer HBV WT and treated with DMSO (lane 2), or transfected by 1.3 mer HBV WT (4 μg) and treated with 20 μM of PiB for 72 h (lane 3). **(B)** HepAD38 was treated with mock (none, lane 1) or DMSO (lane 2), or treated with 20 μM PiB (lane 3) for 72 h. **(C)** HepG2 cells were transfected with mock (lane 1), or 1.3 mer HBV WT (4 μg) transfected and treated with DMSO (lane 2), or 4 μg 1.3 mer HBV WT transfected and treated with PiB (20 μM) for 72 h (lane 3). **(D)** HepG2.2.15 cells were treated with mock (none, lane 2) or treated DMSO (lane 3) or PiB (20 μM) treated for 72 h (lane 4). Lane 1 indicates mock-transfected HepG2 cells (negative control). The lysates were subjected to SDS-PAGE and immunoblotting, NAGE, Northern, and Southern blotting, as described previously (Piracha et al., 2018). The presented data are representative of three independent experiments. The statistical analysis was determined using Student’s *t*-test. *p < 0.05, **p < 0.005, ***p < 0.0005 relative to the control.

Since the competitive irreversible inhibitor of PPIase parvulins, the Juglone, inhibited HBV replication, we speculated that the reversible inhibitor PiB might also affect viral replication in multiple HCC cell lines. The expression of HBc, core particles, and HBV DNA levels were notably abrogated. Since HBc was downregulated, we speculated that PiB-mediated inhibition might affect HBV transcription. Northern blotting revealed that the levels of HBV RNAs were significantly reduced in PiB-treated 1.3 mer HBV-WT transfected Huh7 (Figure 4A), HepAD38 (Figure 4B), 1.3 mer HBV-WT transfected HepG2 (Figure 4C), and HepG2.2.15 (Figure 4D) cells (Figures 4A–C lanes 2 and 3, Figure 4D lanes 3 and 4).

### ATRA-mediated inhibition of PIN1 and PIN4 reduced viral replication

**Figure 5:**
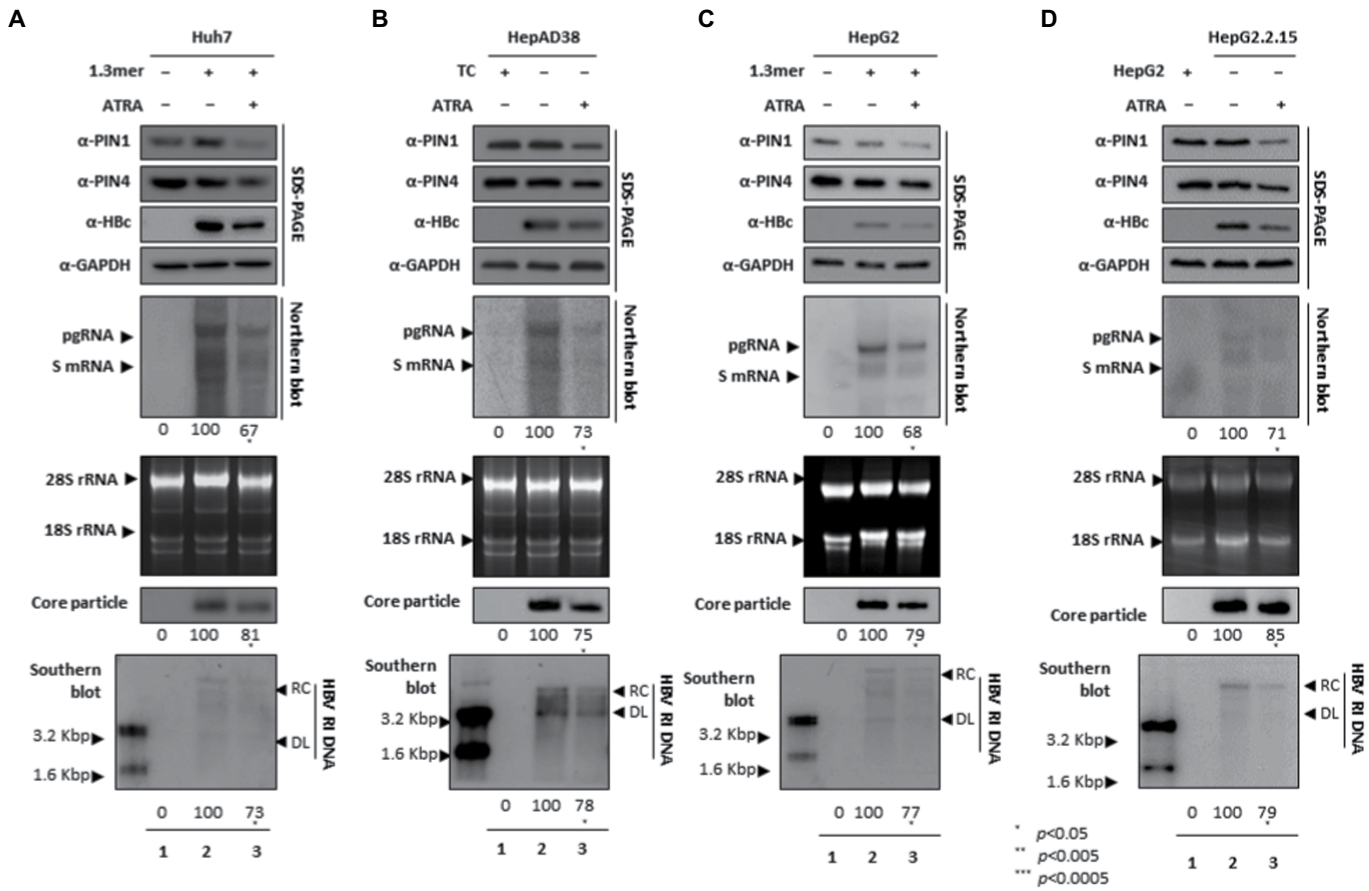
PIN1 and PIN4 inhibition *via* ATRA abrogate HBV replication in Huh7, HepAD38, HepG2, and HepG2.2.15. **(A–D)** ATRA reduces HBV replication. **(A)** The Huh7 cells were mock-transfected (lane 1), transfected with 4 μg of 1.3 mer HBV WT and treated with DMSO (lane 2), or transfected by 1.3 mer HBV WT (4 μg) and treated with 30 μM of ATRA for 72 h (lane 3). **(B)** HepAD38 was treated with mock (none, lane 1) or DMSO (lane 2), or treated with 30 μM ATRA (lane 3) for 72 h. **(C)** HepG2 cells were transfected with mock (lane 1), 1.3 mer HBV WT (4 μg) transfected and treated with DMSO (lane 2), or 4 μg 1.3 mer HBV WT transfected and treated with ATRA (30 μM) for 72 h (lane 3). **(D)** HepG2.2.15 cells were treated with DMSO (lane 2) or ATRA (30 μM) treated for 72 h (lane 3). Lane 1 indicates mock-transfected HepG2 cells (negative control). The lysates were subjected to SDS-PAGE and immunoblotting, NAGE, Northern, and Southern blotting. The presented data are representative of three independent experiments. The statistical analysis was determined using Student’s *t*-test. *p < 0.05, **p < 0.005, ***p < 0.0005 relative to the control.

Since ATRA-mediated inhibition of PIN1 inhibited breast cancer, leukemia, and liver cancer (Kozono et al., 2018). We asked whether ATRA-mediated inhibition of PIN1 and PIN4 affects HBV replication in HCC cells. Of note, upon ATRA treatment, the expression levels of PIN1 and PIN4 were reduced. Congruent with the effects of Juglone and PiB, the ATRA-mediated inhibition of PIN1 and PIN4 also reduced the expression of HBc, HBV RNA transcripts, core particles, and HBV DNA levels in Huh7 (Figure 5A), HepAD38 (Figure 5B), HepG2 (Figure 5C), and HepG2.2.15 (Figure 5D) cells (Figure 5A–D lanes 2 and 3).

### 6,7,4′-THIF-mediated PPIase parvulin inhibition downregulates HBV replication

**Figure 6:**
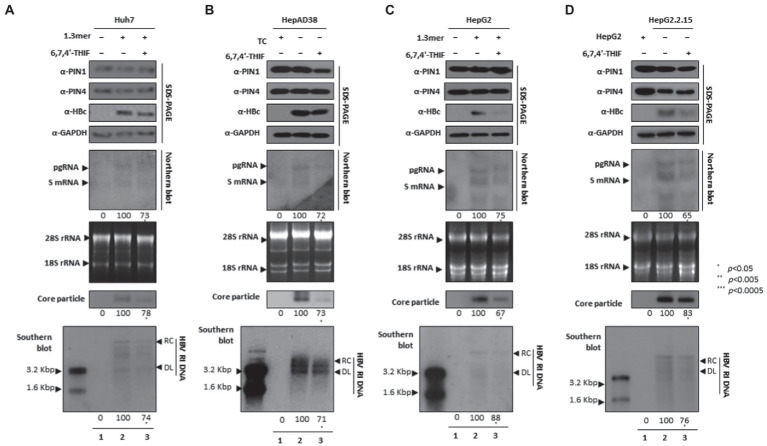
PIN1 and PIN4 inhibition *via* 6,7,4′-THIF reduced HBV replication in Huh7, HepAD38, HepG2, and HepG2.2.15. **(A–D)** 6,7,4′-THIF abrogated HBV replication. **(A)** The Huh7 cells were mock-transfected (lane 1), transfected with 4 μg of 1.3 mer HBV WT and treated with DMSO (lane 2), or transfected by 1.3 mer HBV WT (4 μg) and treated with 30 μM of 6,7,4′-THIF for 72 h (lane 3). **(B)** HepAD38 was treated with mock (none, lane 1) or DMSO (lane 2), or treated with 30 μM 6,7,4′-THIF (lane 3) for 72 h. **(C)** HepG2 cells were transfected with mock (lane 1), 1.3 mer HBV WT (4 μg) transfected and treated with DMSO (lane 2), or 4 μg 1.3 mer HBV WT transfected and treated with 6,7,4′-THIF (30 μM) for 72 h (lane 3). **(D)** HepG2.2.15 cells were treated with DMSO (lane 2) or 6,7,4′-THIF (30 μM) treated for 72 h (lane 3). Lane 1 indicates mock-transfected HepG2 cells (negative control). The lysates were subjected to SDS-PAGE and immunoblotting, NAGE, Northern, and Southern blotting. The presented data are representative of three independent experiments. The statistical analysis was determined using Student’s *t*-test. *p < 0.05, **p < 0.005, ***p < 0.0005 relative to the control.

The 6,7,4′-trihydroxyisoflavone reduced PIN1 PPIase activity, caused increased apoptosis of esophageal cancer, and inhibited proliferation (Lim et al., 2017). In addition, the anticancer activity of 6,7,4′-THIF was also determined in colon cancer (Lee et al., 2011). We speculated that 6,7,4′-THIF-mediated inhibition of PPIase PIN1 and PIN4 might affect HBV replication. The HBc protein expression, HBV transcription (levels of pgRNA, S mRNAs, and X mRNA), core particle synthesis, and HBV DNA replication were significantly aborted in 6,7,4′-THIF-treated HCC cells (Figures 6A–D).

### KPT-6566 restricted HBV DNA replication by targeting PIN1 and PIN4 PPIases

**Figure 7:**
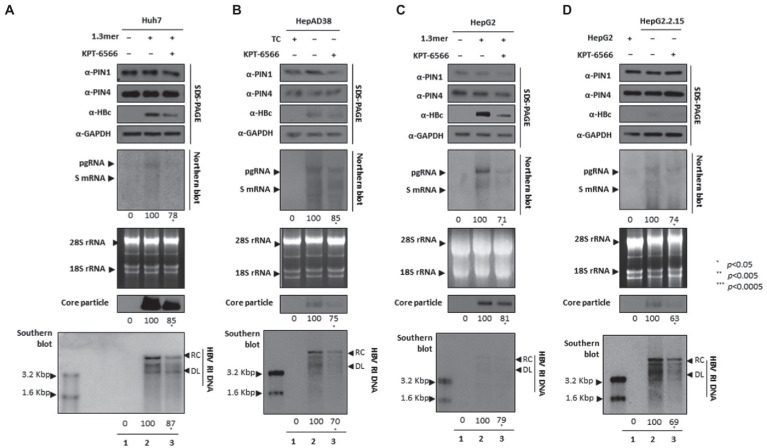
KPT-6566 mediated inhibition of PIN1 and PIN4 lowered HBV replication in Huh7, HepAD38, HepG2, and HepG2.2.15. **(A–D)** KPT-6566 reduced HBV replication. **(A)** The Huh7 cells were mock-transfected (lane 1), transfected with 4 μg of 1.3 mer HBV WT and treated with DMSO (lane 2), or transfected by 1.3 mer HBV WT (4 μg) and treated with 30 μM of KPT-6566 for 72 h (lane 3). **(B)** HepAD38 was treated with mock (none, lane 1) or DMSO (lane 2), or treated with 30 μM KPT-6566 (lane 3) for 72 h. **(C)** HepG2 cells were transfected with mock (lane 1), 1.3 mer HBV WT (4 μg) transfected and treated with DMSO (lane 2), or 4 μg 1.3 mer HBV WT transfected and treated with KPT-6566 (30 μM) for 72 h (lane 3). **(D)** HepG2.2.15 cells were treated with DMSO (lane 2) or KPT-6566 (30 μM) treated for 72 h (lane 3). Lane 1 indicates mock-transfected HepG2 cells (negative control). Cellular lysates were subjected to SDS-PAGE and immunoblotting, NAGE, Northern, and Southern blotting. The presented data are representative of three independent experiments. The statistical analysis was determined using Student’s *t*-test. *p < 0.05, **p < 0.005, ***p < 0.0005 relative to the control.

Since KPT-6566 covalently binds to PIN1, it inhibits PPIase activity and cellular proliferation, therefore exhibiting significant antitumor potential (Campaner et al., 2017). We asked whether KPT-6566-mediated inhibition of PIN1 and PIN4 influenced HBV replication in HCC cells. Consistent with the findings from other parvulin inhibitors (Juglone, PiB, ATRA, and 6,7,4′-THIF), the KPT-6566-mediated inhibition of PIN1 and PIN4 also restricted HBc protein expression, HBV transcriptional activities, core particle synthesis, and HBV DNA replication in KPT-6566-treated Huh7 (Figure 7A), HepAD38 (Figure 7B), HepG2 (Figure 7C), and HepG2.2.15 (Figure 7D) cells (Figures 7A–D lanes 2 and 3).

### EGCG-mediated PIN1 and PIN4 inhibition suppressed HBV replication

**Figure 8:**
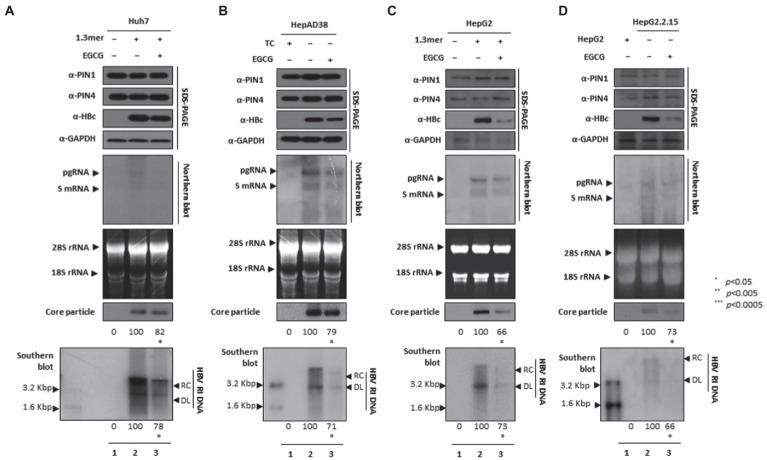
PIN1 and PIN4 inhibition *via* EGCG reduced HBV replication in Huh7, HepAD38, HepG2, and HepG2.2.15. **(A–D)** EGCG reduced HBV replication. **(A)** The Huh7 cells were mock-transfected (lane 1), transfected with 4 μg of 1.3 mer HBV WT and treated with nuclease-free water solvent (lane 2), or transfected by 1.3 mer HBV WT (4 μg) and treated with 40 μM of EGCG for 72 h (lane 3). **(B)** HepAD38 was treated with mock (none, lane 1) or solvent (lane 2), or treated with 40 μM EGCG (lane 3) for 72 h. **(C)** HepG2 cells were transfected with mock (lane 1), 1.3 mer HBV WT (4 μg) transfected and treated with solvent (lane 2), or 4 μg 1.3 mer HBV WT transfected and treated with EGCG (40 μM) for 72 h (lane 3). **(D)** HepG2.2.15 cells were treated with solvent (lane 2) or EGCG (40 μM) treated for 72 h (lane 3). Lane 1 indicates mock-transfected HepG2 cells (negative control). The cellular lysates were subjected to SDS-PAGE and immunoblotting, NAGE, Northern, and Southern blotting. The presented data are representative of three independent experiments. The statistical analysis was determined using Student’s *t*-test. *p < 0.05, **p < 0.005, ***p < 0.0005 relative to the control.

Since EGCG can bind to PIN1 and promote anticancer activities and may induce cancer cell apoptosis (Della Via et al., 2021), we examined whether EGCG-mediated inhibition of PPIases parvulins PIN1 and PIN4 affects HBV replication in HCC cell lines. The HBc, core particle, and HBV DNA levels were reduced significantly. The downregulated HBc expression levels indicated that EGCG-mediated inhibition might affect HBV transcriptional activities. The Northern blotting analysis revealed lower levels of pgRNA, S mRNAs, and X mRNA in drug-treated and 1.3 mer HBV-WT transfected Huh7 (Figure 8A), HepAD38 (Figure 8B), 1.3 mer HBV-WT transfected HepG2 (Figure 8C), and HBV replicating HepG2.2.15 (Figure 8D) cells (Figures 8A–D lanes 2 and 3).

### Parvulin inhibitors abrogated HBV infection by restricting HBV transcription and HBV DNA synthesis

**Figure 9:**
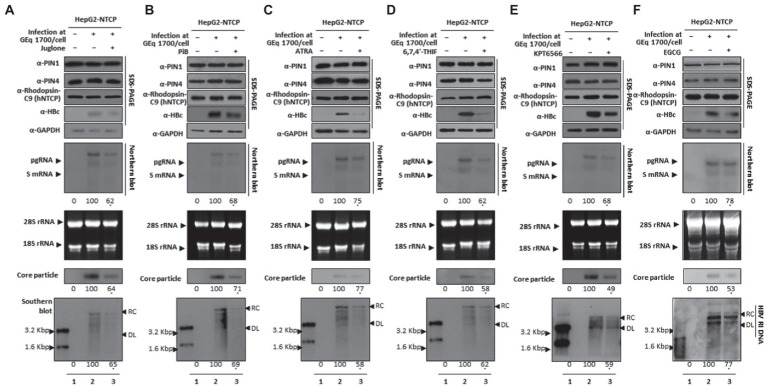
PIN1 and PIN4 Inhibition *via* Juglone, PiB, ATRA, 6,7,4′-THIF, KPT6566, and EGCG abrogate HBV replication in HBV-infected cells. **(A)** Juglone, **(B)** PiB, **(C)** ATRA, **(D)** 6,7,4′-THIF, **(E)** KPT6566, and **(F)** EGCG restricted HBV replication in infected cells. HepG2-hNTCP-C9 cells were seeded and infected, as described previously, in the “Material and Methods” section. Infected cells (lanes 2–3) were treated with solvent (lane 2) or 20 μM Juglone, 20 μM PiB, 30 μM ATRA, 30 μM 6,7,4′-THIF, 30 μM KPT6566, 40 μM EGCG (lane 3) for 9 days, and lysates were subjected to SDS-PAGE and immunoblotting, NAGE, Northern blotting, and Southern blotting. The shown data are representation from three independent experiments. The statistical significance was determined by using Student’s *t*-test. *p < 0.05, **p < 0.005, ***p < 0.0005 relative to the corresponding control.

During the HBV course of infection, HBV attachment and entrance to hepatocytes occur *via* the NTCP receptor. To substantiate the findings that parvulin inhibition restricts HBV replication, we employed HBV infection setting through HBV wildtype virion (prepared from HepAD38 cells) mediated infection into HepG2-hNTCP cells at 1.7 × 10^3^ GEq/cell, as described previously (Bichko et al., 1985; Ko et al., 2014; Nkongolo et al., 2014; Piracha et al., 2018, 2020; Saeed et al., 2019, 2021). HBc, HBV transcripts, core particles, and HBV DNA levels were reduced (Figures 9A–F lanes 2 and 3), indicating the importance of PIN1 and PIN4 during the HBV course of infection. Furthermore, it adds value to the speculation that during HBV infection, inhibition of PIN1 and PIN4 by Juglone, PiB, ATRA, 6,7,4′-THIF, KPT6566, and EGCG might reduce HBV replication or progression toward HCC.

### Juglone, PiB, ATRA, 6,7,4′-THIF, KPT6566, and EGCG did not affect HBV cccDNA levels

**Figure 10:**
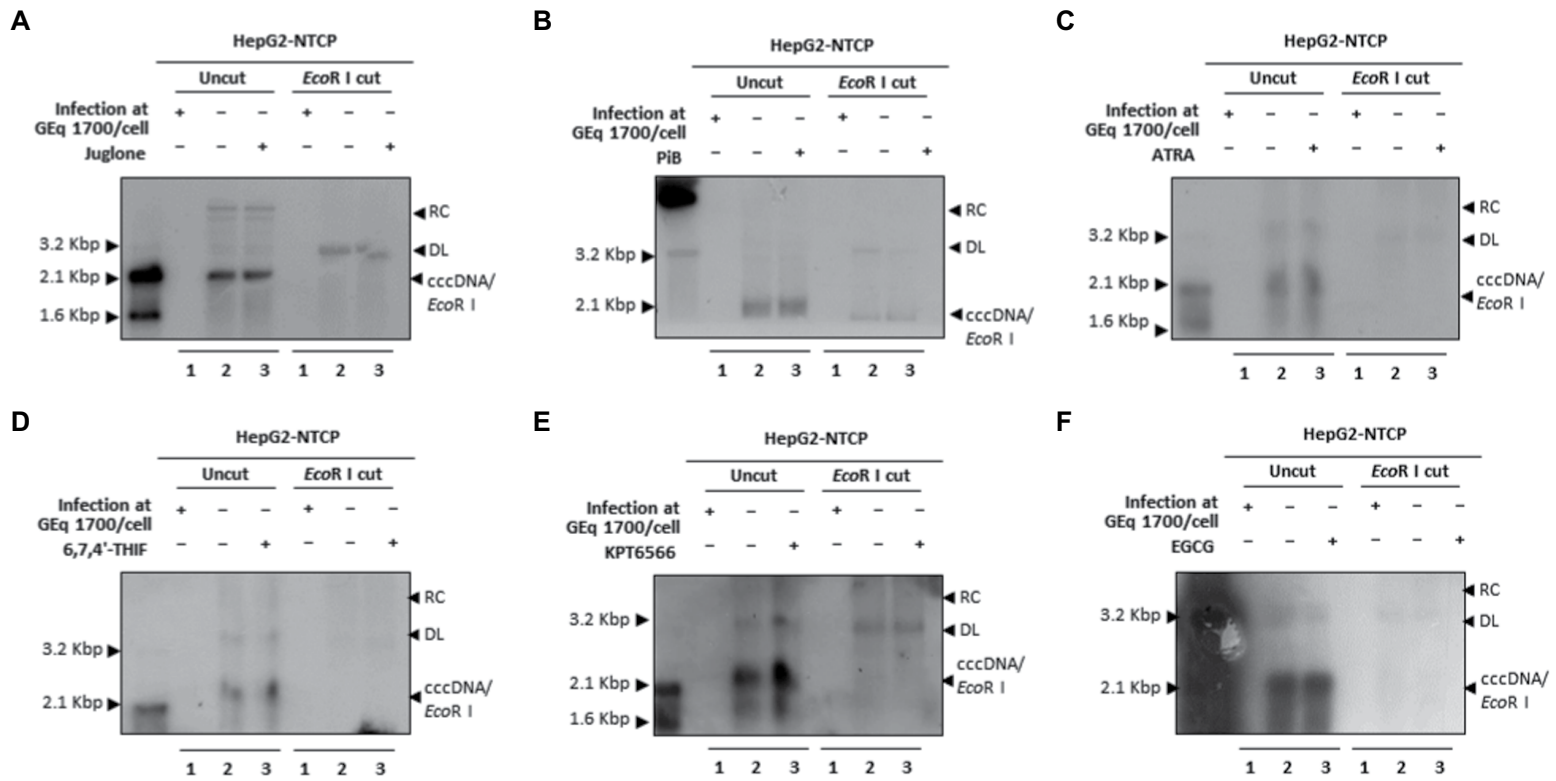
Juglone, PiB, ATRA, 6,7,4′-THIF, KPT6566, and EGCG did not affect HBV cccDNA levels in HBV-infected cells. **(A)** Juglone, **(B)** PiB, **(C)** ATRA, **(D)** 6,7,4′-THIF, **(E)** KPT6566, and **(F)** EGCG parvulin inhibitors did not affect HBV cccDNA levels. Mock-infected HepG2-hNTCP-C9 cells (lane 1) or HBV-infected HepG2-hNTCP-C9 cells (lanes 2–3) were treated with solvent (lane 2) or 20 μM Juglone, 20 μM PiB, 30 μM ATRA, 30 μM 6,7,4′-THIF, 30 μM KPT6566, or 40 μM EGCG (lane 3) for 10 days. HBV cccDNA levels were determined by the Hirt extraction method, as described previously (Saeed et al., 2019). Data are presented from three independent experiments.

Since Juglone, PiB, ATRA, 6,7,4′-THIF, KPT6566, and EGCG abrogated HBV infection and restricted HBV replication (Figure 9), we investigated whether parvulin PPIase inhibitors affect the cccDNA levels during HBV course of infection. The Hirt extraction method was performed to evaluate HBV cccDNA levels, as described previously (Bichko et al., 1985; Piracha et al., 2018, 2020; Saeed et al., 2019, 2021). HBV infection to HepG2-hNTCP-C9 cells followed by 20 μM Juglone (Figure 10A), 20 μM PiB (Figure 10B), 30 μM ATRA (Figure 10C), 30 μM 6,7,4′-THIF (Figure 10D), 30 μM KPT-6566 (Figure 10E), or 40 μM EGCG (Figure 10F) treatment revealed a similar cccDNA synthesis without affecting HBV cccDNA levels.

### Comparative knockdown of PIN1 and PIN4 revealed reduced HBV virion release in the HBV infection system

**Figure 11:**
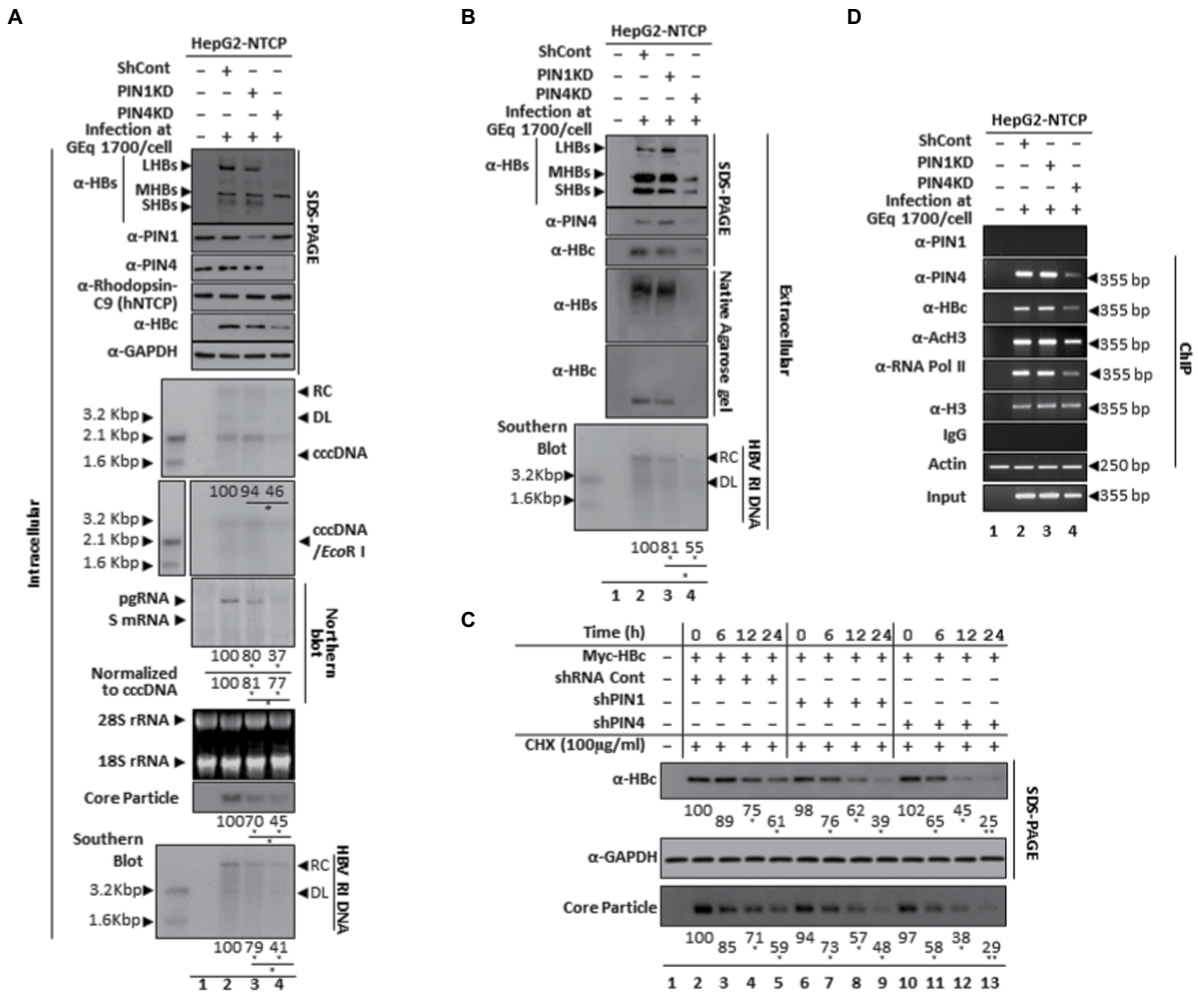
In comparison with PIN1 KD, PIN4 KD further reduced intracellular HBV replication and extracellular virion release and extracellular HBV DNA levels in HBV-infected cells. **(A)** Intracellular HBV replication decreases by PIN1 and PIN4 knockdown. HepG2-hNTCP-C9 (lane 1), HepG2-hNTCP-C9 Sh Cont (lane 2), HepG2-hNTCP-C9-PIN1 KD cells (lane 3), or HepG2-hNTCP-C9-PIN4 KD cells (lane 4) were seeded and mock-infected (lane 1) or HBV infected (lanes 2–3), as described previously (Saeed et al., 2019, 2021). Intracellular lysates were subjected to SDS-PAGE (13.5%) and NAGE immunoblotting, Northern, and Southern blotting. Hirt extraction methods were adopted to examine levels of cccDNA levels in PIN1 KD or PIN4 KD cells. **(B)** Extracellular HBV virion secretion was restricted upon PIN1 or PIN4 knockdown. The HBV virions, naked core particles, HBs subviral particles obtained from non-transduced HepG2-hNTCP-C9 (lane 1), HBV-infected HepG2-hNTCP-C9 Sh Cont (lane 2), HepG2-hNTCP-C9-PIN1 KD cells (lane 3), or HepG2-hNTCP-C9-PIN4 KD cells (lane 4) were examined. SDS-PAGE and immunoblotting were performed using anti-HBs, PIN1, PIN4, and HBc antibodies. **(C)** The stabilities of HBc and core particles were decreased in PIN1 and PIN4 KD Huh7 stable cells. Control shRNA-transduced and PIN1 or PIN4 KD Huh7 cells were transfected with Myc-HBc WT. At 24 h post-transfection, the cells were treated with 100 μg/ml cycloheximide, and lysates were prepared at the indicated time points. **(D)** Chromatin immunoprecipitation (ChIP) was performed in HBV-infected PIN1 KD (lane 2) or PIN4 KD (lane 3) HepG2-hNTCP-C9 cells and subjected to immunoprecipitation with normal rabbit polyclonal IgG or anti-PIN1, anti-PIN4, anti-RNA Pol II, anti-acetyl H3, or anti-H3 antibodies. The immunoprecipitated chromatins were analyzed by PCR. The presented data are representative of three independent experiments. The statistical significance was determined using Student’s *t*-test. *p < 0.05, **p < 0.005, ***p < 0.0005 relative to control.

The comparative effects of PIN1 and PIN4 in the HBV infection system were determined after PIN1 or PIN4 selective knockdown using shRNA lentiviral system. Following HBV infection, the HepG2-hNTCP-C9 was transduced with control shRNA, PIN1 shRNAs, or PIN4 shRNAs (Figure 11A). The results showed that upon PIN1 or PIN4 KD, HBc, HBV transcripts, core particles, intracellular HBV DNA levels, and HBsAg levels were reduced (Figure 11A). Similarly, extracellular HBsAg levels, extracellular HBV virion secretion, extracellular naked core particles, and extracellular HBV DNA levels were significantly downregulated (Figure 11B). Of note, cccDNA levels did not change in PIN1 KD settings; however, the levels were dramatically reduced upon PIN4 KD. In comparison with PIN1 KD, upon PIN4 KD, the HBV replication and HBV virion release were decreased even more, indicating the importance of PIN4 over PIN1 during HBV replication. In comparison with the PIN1 KD, upon PIN4 KD, the stability of HBc and core particles was significantly reduced, indicating that besides PIN1, PIN4 is relatively more critical for the stability of HBc and core particles (Figure 11C).

### PIN1 and PIN4 KD followed by Juglone, PiB, ATRA, 6,7,4′-THIF, KPT6566, and EGCG treatment restricted HBc recruitment to cccDNA, transcriptional activation, and consequently HBV replication

Since PIN1 or PIN4 KD significantly reduced HBc protein expression and HBV transcription, we investigated whether PIN1 or PIN4 KD affects the recruitment of HBc protein to HBV cccDNA. Chromatin immunoprecipitation (ChIP) was performed in HBV-infected HepG2-hNTCP-C9 PIN1 or PIN4 KD cells to determine the comparative effects of these parvulins on cccDNA (Figure 11D). The chromatins were prepared and immunoprecipitated with IgG or anti-PIN1, HBc, PIN4, acetylated H3 (anti-AcH3), RNA polymerase-II (anti-RNA Pol-II), and histone H3 antibodies and furthermore analyzed by semiquantitative PCR (GeneAmp PCR 2700, Applied Biosystems; Bichko et al., 1985; Saeed et al., 2019, 2021; Piracha et al., 2020). In contrast to endogenous PIN1, the endogenous PIN4 was recruited to HBV cccDNA. Of note, upon PIN1 KD, the recruitment of HBc protein to the cccDNA remains unchanged. However, upon PIN4 KD, the recruitment of HBc, anti-RNA Pol II, and anti-AcH3 to the cccDNA was dramatically reduced, indicating that in contrast to PIN1, PIN4 plays significantly more important roles in augmenting HBV replication.

**Figure 12:**
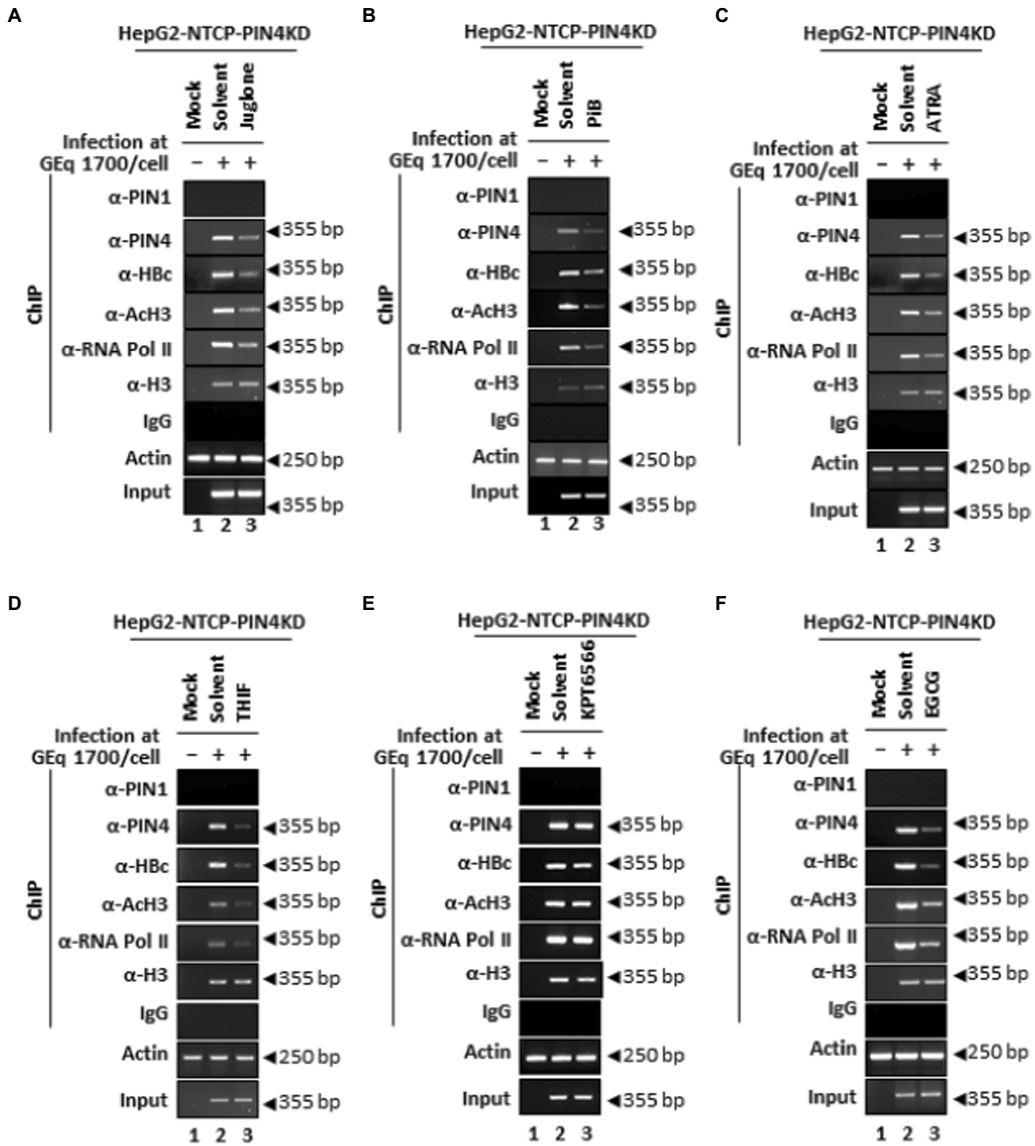
PIN4 KD followed by Juglone, PiB, ATRA, 6,7,4′-THIF, KPT6566, and EGCG treatment significantly reduced HBc recruitment to cccDNA and HBV transcriptional activities in HBV-infected cells. **(A–F)** HepG2-hNTCP-C9-PIN4 KD cells were mock-infected (lane 1) or HBV infected (lanes 2–3) and treated with solvent (lane 2), or parvulin inhibitors **(A)** 20 μM Juglone, **(B)** 20 μM PiB, **(C)** 30 μM ATRA, **(D)** 30 μM 6,7,4′-THIF, **(E)** 30 μM KPT6566, and **(F)** 40 μM EGCG. The chromatin solutions and ChIP were performed in HBV-infected HepG2-hNTCP-C9-PIN4 KD cells, as described in the “Material and Methods” section and were subjected to immunoprecipitation with normal rabbit polyclonal IgG, anti-PIN1, anti-PIN4, anti-RNA Pol II, anti-acetyl H3, or anti-H3 antibodies. The immunoprecipitated chromatins were analyzed by PCR. The presented data are representative of three independent experiments.

Furthermore, among HBV-infected PIN4 KD cells, the effects of Juglone (Figure 12A), PiB (Figure 12B), ATRA (Figure 12C), 6,7,4′-THIF (Figure 12D), KPT6566 (Figure 12E), and EGCG (Figure 12E) were determined followed by ChIP. The results revealed that recruitments of endogenous PIN4 and HBc onto HBV cccDNA were reduced upon treatment with parvulin inhibitors Juglone (Figure 12A), PiB (Figure 12B), ATRA (Figure 12C), 6,7,4′-THIF (Figure 12D), KPT6566 (Figure 12E), and EGCG (Figure 12E). The transcriptional activity of chromatin (anti-RNA Pol II and anti-AcH3-specific Abs) was further reduced by parvulin inhibitor treatment. Of note, PIN4 KD cells further decreased upon treatment with the aforementioned parvulin inhibitors (Figures 12A–D lane 2 vs. 3). PIN1 KD and/or PIN4 KD along with parvulin inhibition *via* Juglone, PiB, ATRA, 6,7,4′-THIF, KPT6566, and EGCG might indicate possible therapeutic options to functionally cure CHB for preventing hepatocarcinogenesis.

## Discussion

Viruses have adopted multiple strategies to dampen host defense mechanisms by modulating several cellular proteins assisting viral replication. Human PPIases (acting as molecular switches) twist the target protein backbone (by cis/trans conformation) for functional activation or deactivation of enzymes (Lu et al., 2007). Viruses use certain host PPIases (cyclophilins, FK506-binding proteins, parvulins, and protein Ser/Thr phosphatase 2A (PP2A) activator) for viral protein modifications supporting their replications (Gothel and Marahiel, 1999; Ünal and Steinert, 2014). The HBV life cycle has been extensively studied; however, several host factors that are involved in viral replication await further investigation. Among the parvulins, PIN1 interacts with the HBx SP motif for HBx transactivation and enhances hepatocarcinogenesis (Pang et al., 2007), while PIN4-encoded Par14/Par17 binds to RP motifs on HBx and upregulates HBV replication (Saeed et al., 2019, 2021). The PIN1 can bind to HBc at the Thr160-Pro and Ser162-Pro motifs and stabilizes it in a phosphorylation-dependent manner to enhance HBV propagation (Pang et al., 2007). HBV core particle has 180 or 240 PIN4 binding sites (*via* single RP motif on HBc). The parvulin-binding RP motif is located in the N-terminal domain, which is critical for core particle assembly (Gallina et al., 1989; Birnbaum and Nassal, 1990). We recently reported that PIN4 binds to both HBc and HBV core particles *via* RP motif at HBc and stabilizes it to support HBV replication (Saeed et al., 2021). Previously, PIN1 inhibition *via* Juglone, PiB, ATRA, 6,7,4′-THIF, KPT6566, and EGCG had been widely examined in various cancers. However, PIN1 inhibition *via* the aforementioned inhibitors was never examined in the context of HBV biology. We first demonstrated PIN1 and PIN4 inhibitory effects *via* Juglone, PiB, ATRA, 6,7,4′-THIF, KPT6566, and EGCG on HBV infection. Herein, we provide evidence suggesting that inhibition of PIN1 and PIN4 *via* parvulin inhibitors can significantly abrogate HBc expression, viral transcription, core particle formation, and HBV virion release.

PIN1-and PIN4-encoded proteins have differential effects on HBV replication. PIN1 can stabilize HBV core protein, promote replication, and might play an important role in HBV-induced HCC (Nishi et al., 2020). PIN4-encoded Par14 and Par17 can bind to HBc and HBV core particles, bind physically to cccDNA, and act as a bridge between HBx-cccDNA and HBc-cccDNA (Saeed et al., 2019, 2021). Herein, through a series of experiments, it was shown that in comparison with PIN1, PIN4 plays more significant and profound effects in upregulating HBV replication starting from cccDNA to virion release. PIN4 knockdown reduced the recruitment of Par14 and Par17 proteins onto the cccDNA and potentially lowered bindings with cccDNA, consequently reducing HBV viral transcription as shown by the Northern blot in HBV-infected HepG2-NTCP. The potential effects of PIN4 binding to HBc and HBV core particles were also extensively investigated, where we have shown that the highly conserved RP site in HBc plays a vital role in physical bindings with PIN4-encoded proteins (Saeed et al., 2021).

Except for PIN1 and PIN4 inhibition *via* parvulin inhibitors, we specifically knocked down PIN1 and PIN4 to examine the exclusive effects of these parvulins on HBV replication. Both PIN1 KD and PIN4 KD significantly reduced HBV replication from viral transcription to virion release. In comparison with PIN1 KD, upon PIN4 KD, the HBc expression and stability, HBV cccDNA levels, HBV transcriptional activities, core particle synthesis and stability, intracellular HBV DNA replication, intracellular HBsAg levels, extracellular HBV virion, extracellular naked core particles, and extracellular HBV DNA levels were abrogated more profoundly, which indicates that PIN4 is more critical than PIN1 in promoting HBV replication. Microtubules are critical for efficient core particle formation and HBV replication (Iwamoto et al., 2017). PIN1 and PIN4 can interact with tubulin to promote its polymerization (Hamdane et al., 2006; Thiele et al., 2011). Due to the limitation of our study, we could not determine whether PIN1 and PIN4 promote HBV replication *via* enhanced tubulin polymerization; however, the evidence adds weightage to our findings that PIN1 and PIN4 efficiently upregulate HBV replication and viral life cycle.

HBV HBc and HBx can influence cccDNA epigenetic dynamics (Hong et al., 2017). Previously, it was speculated that HBc constitutes repressive effects on HBV transcription by compacting cccDNA (Bock et al., 2001). However, several recent studies are supported by the evidence that HBc can preferentially interact with cccDNA at II CpG island, tends to transform the spacing of nucleosomes at cccDNA-bound histone complex, and is capable of triggering hypomethylation of histones. This phenomenon further progresses toward augmented recruitment levels of cyclic AMP-responsive enhancer binding protein and increases histone acetylation, therefore promoting cccDNA transcription (Guo et al., 2011). Many host proteins can directly bind to cccDNA and enhance transcription (Mohd-Ismail et al., 2019). Of note, in contrast to PIN4, PIN1 did not show direct binding to the cccDNA. However, in contrast to PIN4 KD (where cccDNA levels and HBc recruitment to cccDNA were significantly downregulated), upon PIN1 KD, the levels of cccDNA and HBc recruitment to cccDNA remained unchanged. Interestingly, upon PIN4 KD, followed by drug (Juglone, PiB, ATRA, 6,7,4′-THIF, KPT6566, or EGCG) treatment, HBc recruitment to cccDNA and consequently HBV transcriptional activities decreased dramatically. Given that the upregulated HBsAg and HBV DNA levels are linked to a high risk of development of HCC (Tseng et al., 2012; Gai et al., 2013; Kawanaka et al., 2014), PIN1 plays important roles in tumor development (Kozono et al., 2018); PIN4 is critical for PGCIα induction and tumor progression (Frattini et al., 2018); and inhibition or knockdown of PIN1 and PIN4 significantly reduced HBV DNA and HBsAg levels. It can be inferred that PIN1 and PIN4 (preferably) knockdown and/or parvulin inhibition might be important therapeutic options to functionally cure CHB and/or HBV-associated HCC.

## Materials and methods

### Cell culture and DNA transfection

PLC/PRF5, SNU368, SNU387, Chang, and SNU449 cells were grown in DMEM, and human liver epithelial THLE-2 cells (ATCC®CRL-2706™) were grown in bronchial epithelial cell basal medium (BEBM; Lonza #CC-3171) with 10% FBS (Gibco) and 1% penicillin–streptomycin (PS). The base medium for THLE-2 is ATCC-recommended essential media supplemented with BPE, hydrocortisone, hEGF, insulin, triiodothyronine, transferrin, retinoic acid, phosphoethanolamine, and human recombinant EGF. Huh7, HepAD38, HepG2, HepG2.2.15, HepG2-hNTCP-C9, and HEK293T cells were grown in DMEM with 10% FBS and 1% PS at 37°C with 5% CO_2_. G418 (0.5 mg/ml) was added for selection in HepG2.2.15 cells. HepAD38 cells were maintained in 1 μg/ml of tetracycline (TC), and the medium was replenished with fresh medium (without TC) to initiate HBV replication. HEK293T cells were passaged on the second day; however, Huh7, HepAD38, HepG2, and HepG2.2.15 cells were passaged on every third day. However, for infection-related experiments, the viral inoculum was harvested from the supernatant of HepAD38 cells (Saeed et al., 2019). The HBV surface antigen possesses group-specific antigenic determinant ‘a’ and two pairs of mutually exclusive subtype-specific determinants named “d” or “y” and “r” or “w”; hence, it can be further distinguished into four major subtypes including adr, adw, ayw, and ayr (Bichko et al., 1985).

Notably, 3 × 10^6^ of HepAD38 cells in a 6 cm plate were seeded in TC media, and the following day, the media was replaced with fresh medium (without TC). Of note, 3 × 10^6^ of HepG2.2.15 cells were seeded in 6 cm plates; 3 × 10^6^ of HepG2 cells were transfected with 4 μg of 1.3 mer HBV WT ayw with 12 μg/μl PEI dissolved in 200 μl of Opti-MEM. Similarly, 2 × 10^6^ of Huh7 cells were transfected with 4 μg of 1.3 mer HBV WT and 12 μg/μl of PEI in 200 μl of Opti-MEM.

### Establishment of stable cell lines

Short hairpin RNAs (shPIN1 and shPIN4, shControl) in the pLK0.1 vector form were purchased from Sigma Aldrich USA (SHCLNG-NM_006223 and SHC001). To generate PIN1 or PIN4 knockdown stable cells, 1 × 10^6^ of HEK293T cells in a 6 cm plate were triple-transfected with 1.5 μg of pGAG-Pol, 0.5 μg of pVSV-G, 2 μg of pLK0.1-shControl, and 2 μg of pLK0.1-shPIN1 or shPIN4. Afterward, pseudoviral particles having shPIN1, shPIN4, or shControl RNAs were inoculated onto HepG2-hNTCP-C9 cells and selected with puromycin to generate PIN1 or PIN4 KD stable cells. After 24 h, the cellular media was replenished with a new medium, and supernatant (containing lentivirus pseudoviral particles with PIN1 or PIN4 KD transcripts) was harvested at 72 h post-transfection. A volume of 2 ml of the collected supernatant and 10 μg/ml polybrene (hexadimethrine bromide, Sigma-Aldrich) in the fresh 2 ml of medium were mixed and transduced to HepG2-hNTCP-C9 cells. At 24 h post-inoculation, the cells were replaced with fresh medium, grown until more than 90% confluency, diluted with 1:2 or 1:3, and initiated for selection by puromycin (10 μg/ml).

### HBV capsid or core particle isolation and immunoblotting

HBV core particles were analyzed using 50 mM NaCl, 10 mM Tris–HCl [pH 8.0], and 1 mM EDTA with 0.2% NP-40 (Sigma-Aldrich, United States) cell lysis buffer. The lysate (4%) was subjected to 1% NAGE and proteins were shifted to polyvinylidene fluoride (PVDF) membranes (Millipore). Immunoblotting *via* primary anti-HBc (rabbit polyclonal of 1:1,000 dilution) antibodies bound to secondary (anti-rabbit of 1:5,000 dilution) antibodies was visualized through Western blotting detection reagents and enhanced chemiluminescence (ECL, Amersham). ImageJ. 1.46r revealed relative intensities of HBV core particles, as described previously (Piracha et al., 2018).

### Cell cytotoxicity assay

The cytotoxic effects of parvulin inhibitors (Juglone, PiB, ATRA, 6,7,4′-THIF, KPT6566, and EGCG) *via* MTT (3-[4,5-dimethylththiazol-2-yl]-2,5-diphenyltetrazolium bromide) assay were determined in Huh7, HepAD38, HepG2, and HepG2.2.15 cell lines. The cells were grown in 96-well microplates and incubated with serial dilutions of Juglone, PiB, ATRA, 6,7,4′-THIF, KPT6566, or EGCG for 48 h at 37°C. The cellular viabilities were examined after replacing the culture medium with 100 μl of MTT in DMEM. After 3 h, 100 μl of DMSO was added to dissolve the formazan of MTT. The absorbance value was measured at 570 nm *via* plate reader. Afterward, the concentrations of Juglone, PiB, ATRA, 6,7,4′-THIF, KPT6566, and EGCG at which cell viability was reduced to 50% (CC50) to that of the control were determined.

### Sodium dodecyl sulfate-polyacrylamide gel electrophoresis and immunoblotting

The Bradford assay was performed to determine equal protein levels in cellular lysates, as described previously (Piracha et al., 2018). The lysates (0.2% NP-40-NTE) were subjected to SDS-PAGE (13.5%) and immunoblotting using anti-PIN1 (mouse monoclonal at 1:1,000 dilution, Santa Cruz Biotech # sc-46660), anti-PIN4 (rabbit monoclonal at 1:1,000 dilution, Abcam #ab155283), anti-GAPDH (mouse monoclonal at 1:5,000 dilution, Santa Cruz #sc-32,233), anti-H3 (rabbit polyclonal at 1:5,000, Abcam #ab1791), anti-VDAC (mouse monoclonal at 1:1,000 dilution, Calbiochem #529532), anti-HBs (rabbit polyclonal at 1:1,000 dilution, Virostat #GF528), and anti-C9 (mouse anti-rhodopsin monoclonal at 1:1,000, Millipore#MAB5356) followed by anti-rabies or anti-mouse antibodies (at 1:5,000 dilution) coupled to horseradish peroxidase. The immunoblots were visualized using ECL, and relative protein levels were determined using ImageJ. 1.46r.

### Northern and southern blotting and autoradiography

HBV RNA and DNA syntheses were determined in HBV replicating cells using Northern and Southern blotting methods, as described previously (Piracha et al., 2018). Briefly, total RNAs were extracted according to the manufacturer’s instructions. A volume of 20 μg of RNAs was denatured for 10 min at 65°C and subjected to 1.2% NAGE (using ultrapure agarose, Invitrogen #16500500) with buffer containing 10 mM EDTA, 200 mM MOPS, (1 × MOPS), 50 mM sodium acetate (pH 7.0), and formaldehyde (Sigma-Aldrich #F8775). Afterward, the HBV RNAs were shifted to a nylon membrane (Roche #11417240001) for HBV full-length specific, ^32^P-labeled random-primed probe-labeled hybridization at 68°C (for 4 h) and autoradiography. Similarly, by Southern blotting, the extracted DNAs from core particles or capsids were separated by NAGE, shifted to a nylon membrane (Whatman #10416296), ^32^P-labeled random-primed probe mediately hybridized for autoradiography, as described previously (Bichko et al., 1985; Piracha et al., 2018, 2020; Saeed et al., 2019, 2021).

### HBV virion analysis

To analyze HBV virion, 6 × 10^6^ of HepAD38-Par14/17 cells in a 10 cm plate were seeded and TC was removed at 24 h post-seeding. Four days after TC removal, cells were harvested and culture supernatants were collected. Supernatants were cleared by centrifugation followed by filtration through a 0.45 μM syringe-top filter, layered onto 20% sucrose cushion in TNE buffer, and subjected to ultra-centrifugation (Beckman Coulter Optima L-90 K) at 26,000 rpm (for 3 h at 4°C). The pellet having naked core particles, HBs subviral particles, and HBV virions were re-suspended in NTE buffer and subjected to 1% NAGE, 13.5% SDS-PAGE, and Southern blotting, as described previously (Saeed et al., 2019, 2021).

### Nuclear, cytoplasmic, and mitochondrial cellular fractionation

Notably, 3 × 10^6^ of HepG2 cells in a 6 cm plate were harvested at 72 h. The cellular nuclear and cytoplasmic fractionations were prepared, as described previously (Saeed et al., 2019, 2021). The cells were harvested in a 0.5% 0.1 mM EDTA, 0.5 M sucrose, 10 mM HEPES [pH 7.9], Triton X-100, 10 mM NaF, 50 mM NaCl, and 1 mM DTT containing buffer. After 5 min, the lysates were centrifuged for 10 min at 100 × *g*.

### The nuclear fraction in the pellet was separated from cytoplasmic fractions

The pellet was washed with 500 μl of buffer containing 1 mM DTT, 10 mM HEPES [pH 7.9], 0.1 mM EDTA, 10 mM KCl, and 0.1 mM EGTA. The nuclear pellet was lysed with 300 μl of nuclear lysis (500 mM NaCl, 1 mM DTT, 10 mM HEPES [pH 7.9], 0.1 mM EDTA, 0.1 mM EGTA, and 0.1% NP-40) buffer. The supernatants were centrifuged for 10 min at 4°C at 13,000 × *g*, the debris was removed, and supernatants were collected as nuclear fractionation extract. From the cytoplasmic fractionation, the mitochondrial fractions were separated, as described previously (Saeed et al., 2019). Briefly, the cytoplasmic supernatants were subjected to differential centrifugation for 30 min at 4°C at 10,000 × *g*. The pellets obtained were isolated as a mitochondrial fraction. Again, the separated supernatant was re-centrifuged for 15 min at 4°C at 13,000 × *g* and collected as cytoplasmic fractionation. Isolated mitochondria were lysed using a buffer containing 150 mM NaCl, 50 mM Tris–HCl [pH 7.4], 0.5% (v/v) Triton X-100, 1 mM phenylmethanesulfonyl fluoride (PMSF), 2 mM EGTA, 2 mM EDTA, and 1 × protease inhibitor cocktail (Calbiochem, 535,142) for 5 min. The collected suspension was centrifuged for 10 min at 10,000 × *g*, debris was removed, and the supernatants were collected as mitochondrial fractions.

### Preparation of HBV virion and conduct of HBV infection

HBV inoculum was harvested from HepAD38 cells for infection experiments. HepAD38 cells in DMEM plus 10% FBS, 1% PS, G418 (0.5 mg/ml), insulin (5 μg/ml), and hydrocortisone hemisuccinate (50 μM) were grown, and on every third day after passage of day 10^th^ (till 31^st^), the media supernatants were collected and concentrated *via* 20 to 60% sucrose gradient by ultra-centrifugation (Beckman Coulter Optima L-90 K) method, as described previously (Saeed et al., 2019, 2021). The HBV DNAs from the pelleted precipitant were examined by Southern blotting. Notably, 2 × 10^5^ of HepG2-hNTCP-C9, HepG2-hNTCP-C9-shControl, HepG2-hNTCP-C9-shPIN1, or HepG2-hNTCP-C9-shPIN4 cells were seeded in 6-well plates (Corning#354249) and infected with HBV at 1.7 × 10^3^ GEq per cell in media containing 4% PEG (Affymetrix#25322-68-3). The following day, the cell media was refreshed after PBS washing and maintained in 2.5% DMSO containing media. On the 9^th^ day post-infection, the cellular lysates were prepared and subjected to SDS-PAGE and immunoblotting, core particle immunoblotting, and Southern blot analysis. At 5 days post-infection, HBV RNA levels were determined by Northern blotting.

### Juglone, PiB, ATRA, 6,7,4′-THIF, KPT6566, or EGCG treatment to HBV replicating cells

Juglone (Sigma Aldrich, AG17724), PiB (Calbiochem, CAS 64005–90-9), ATRA (Sigma-Aldrich, R2625), 6,7,4′-THIF (Chem Faces, CFN90796), KPT6566 (ProbeChem, PC-61182), or EGCG (Sigma Aldrich, 989–51-5) was treated to Huh7, HepAD38, HepG2, HepG2.2.15, and HepG2-hNTCP-C9 cells. Prior to the conduct of experiments, the cytotoxic effects of Juglone, PiB, ATRA, 6,7,4′-THIF, KPT6566, or EGCG were examined *via* MTT assay (3-[4,5-dimethylththiazol-2-yl]-2,5-diphenyltetrazolium bromide), as described previously (Bichko et al., 1985; Piracha et al., 2018, 2020; Saeed et al., 2019, 2021). Briefly, selected HCC cell lines (HepG2, Huh7, HepG2.2.15, and HepAD38) in 6 cm plates were treated with Juglone (20 μM), PiB (20 μM), ATRA (30 μM), 6,7,4′-THIF (30 μM), KPT6566 (30 μM), or EGCG (40 μM) at transfection or at the time of TC removal from HepAD38 cells. Juglone (20 μM in ethanol, Sigma Aldrich, AG17724) was treated to 1.3 mer HBV-WT transfected Huh7 cells (Figure 3A) or TC-depleted HepAD38 cells (Figure 3B) or to 1.3 mer HBV-WT transfected HepG2 (Figure 3C) or HepG2.2.15 (Figure 3D) cells for 72 h. PiB (20 μM in DMSO, Calbiochem, CAS 64005-90-9) was treated for 72 h to 1.3 mer HBV-WT transfected Huh7 cells (Figure 4A) or TC-depleted HepAD38 cells (Figure 4B) or to 1.3 mer HBV-WT transfected HepG2 cells (Figure 4C) or HepG2.2.15 (Figure 4D) cells. To examine the effects on HBV replication, ATRA (30 μM in DMSO, R2625 Sigma-Aldrich) was treated for 72 h to 1.3 mer HBV-WT transfected Huh7 cells (Figure 5A) or TC-depleted HepAD38 cells (Figure 5) or to 1.3 mer HBV-WT transfected HepG2 cells (Figure 5C) or HBV replicating HepG2.2.15 (Figure 5D) cells. 6,7,4′-THIF (30 μM in DMSO, CFN90796 Chem Faces) was treated for 72 h to 1.3 mer HBV-WT transfected Huh7 cells (Figure 6A) or TC-depleted HepAD38 cells (Figure 6B) or to 1.3 mer HBV-WT transfected HepG2 cells (Figure 6C) or HBV replicating HepG2.2.15 (Figure 6D) cells. KPT-6566 (30 μM in DMSO, PC-61182 ProbeChem) was treated for 72 h to 1.3 mer HBV-WT transfected Huh7 cells (Figure 7A) or TC-depleted HepAD38 cells (Figure 7B) or to 1.3 mer HBV-WT transfected HepG2 cells (Figure 7C) or HBV replicating HepG2.2.15 (Figure 7D) cells. A volume of 40 μM EGCG in nuclease-free water (989-51-5, Sigma Aldrich) was treated for 72 h to 1.3 mer HBV-WT transfected Huh7 cells (Figure 8A) or TC-depleted HepAD38 cells (Figure 8B) or to 1.3 mer HBV-WT transfected HepG2 cells (Figure 8C) or HepG2.2.15 (Figure 8D) cells. At 72 h post-treatment, the cellular lysates were prepared, as described previously (Saeed et al., 2019). Following infection to 2 × 10^5^ HepG2-hNTCP-C9 cells, 20 μM Juglone (Figure 9A), 20 μM PiB (Figure 9B), 30 μM ATRA (Figure 9C), 30 μM 6,7,4′-THIF (Figure 9D), 30 μM KPT-6566 (Figure 9E), or 40 μM EGCG (Figure 9F) was treated for 9 days.

### HBc stability analysis

PIN1 KD or PIN4 KD Huh7 (2 × 10^5^) cells were grown on 6-well plates and transfected with 0.5 μg of Myc-HBc with 2 μg/ml of PEI in 100 μl of Opti-MEM. After 24 h, the medium was replenished with fresh media containing 100 μg/ml cycloheximide (Sigma #C1988-1G). After 0, 6, 12, and 24 h post-treatment, the cells were harvested and lysates were subjected to SDS-PAGE, Western blotting, and native agarose gel electrophoresis and immunoblotting.

### cccDNA analysis and chromatin immunoprecipitation

HBV cccDNA synthesis was examined using the protein-free Hirt cccDNA extraction method. Briefly, HBV-infected PIN1 or PIN4 KD cells at approximately 100% confluency were incubated for 10 days, lysed with 10 mM EDTA and 10 mM Tris–HCl [pH 7.5] containing 0.6% SDS-TE buffer, treated for 16 h by adding 5 M NaCl to the final concentration of 1 M, and precipitated by centrifugation for 30 min at 14,500 × *g*. The supernatants were subjected to multiple phenol extractions (two times) followed by cccDNA extraction *via* phenol/chloroform. The supernatant containing cccDNA was precipitated by ethanol and furthermore analyzed by Southern blotting. The HBV cccDNA ChIP was performed using cccDNA-specific primers and analyzed by PCR (GeneAmp 2,700, Applied Biosystems), as described previously (Bichko et al., 1985; Piracha et al., 2018, 2020; Saeed et al., 2019, 2021). From the clinical specimens, HBV cccDNA levels were examined, as described previously (Zhang et al., 2017), using 251F 5′-GAC TYG TGG TGG ACT TCT C-3′ and 1190R 5′-TCA GCA AAY ACT YGG CA-3′ set of primers.

### Statistical analysis

The presented data are shown as the mean ± standard deviation. The mean values were compared by using Student’s *t*-test. GraphPad Prism version 5 (GraphPad Software) was used for graphs. *p*-values of <0.05 are considered statistically significant.

## Data availability statement

The original contributions presented in the study are included in the article/supplementary material, further inquiries can be directed to the corresponding author.

## Ethics statement

The studies involving human participants were reviewed and approved by International Center of Medical Sciences Research Islamabad Pakistan. The patients/participants provided their written informed consent to participate in this study.

## Author contributions

US conceived the study, performed all experiments, analyzed the data, and wrote the manuscript. ZP assisted US in performing experiments and analysis of data and critically reviewed the study. All authors contributed to the article and approved the submitted version.

## Conflict of interest

The authors declare that the research was conducted in the absence of any commercial or financial relationships that could be construed as a potential conflict of interest.

## Publisher’s note

All claims expressed in this article are solely those of the authors and do not necessarily represent those of their affiliated organizations, or those of the publisher, the editors and the reviewers. Any product that may be evaluated in this article, or claim that may be made by its manufacturer, is not guaranteed or endorsed by the publisher.
